# A multicenter, randomized, double-blind, placebo-controlled trial evaluating the efficacy and safety of Taoren Honghua Jian granule in patients with stable coronary artery disease

**DOI:** 10.1097/MD.0000000000017753

**Published:** 2019-11-01

**Authors:** Yiru Wang, Yiyi Zhang, Yiyue Du, Ying Yang, Jing Wei, Na Zhang, Meijiao Mao, Wenting Du, Ping Liu

**Affiliations:** a Longhua Hospital affiliated to Shanghai University of Traditional Chinese Medicine; b Department of Traditional Chinese Medicine, Gongli Hospital; c Shanggang Community Health Service Center, Shanghai Pudong New Area; d Department of Traditional Chinese Medicine, Shanghai Xuhui Central Hospital, Shanghai, China.

**Keywords:** randomized controlled trial, stable coronary artery disease, Taoren Honghua Jian granule, traditional Chinese medicine

## Abstract

**Introduction:**

Stable coronary artery disease (SCAD) is a common clinical style in patients with coronary artery disease. Inflammation and immune injury in SCAD have been proven. Current interventions can relieve patients’ symptoms, but none are stable to improve the immune damage significantly. Chinese herb is used to treat stable angina pectoris as an alternative therapy. Taoren Honghua Jian granule (THJG) is a classical formula from which patients can benefit, but lack convincing evidence. Therefore, we report a protocol of high-quality randomized controlled trial.

**Methods/design:**

We will conduct a randomized, double-blind, placebo-controlled clinical trial to assess the effect of THJG in relief of symptoms and blood immune indexes. A total of 80 patients with SCAD will be enrolled, and treated with THJG or placebo for 4 weeks. The primary outcome measurement is change of integral traditional Chinese medicine (TCM) syndrome score from baseline to 4 weeks’ treatment. The 2nd outcome measurements include change of integral TCM syndrome scores from baseline to 2 weeks’ treatment and another 4 weeks’ follow-up, inflammation indexes at baseline and 4 weeks’ treatment. It also includes Seattle Angina Questionnaire, major adverse cardiovascular events, Athens Insomnia scale, and 36-item short form health survey score from baseline to 2 weeks’ and 4 weeks’ treatment, and another 4 weeks’ follow-up. Adverse events will be evaluated throughout the trial.

**Discussion:**

The results of this trial will prove whether THJG could alleviate symptoms, control inflammation, and improve quality of life in patients with SCAD.

**Trial registration:**

ChiCTR1900021772, registered 8 March, 2019.

## Abstract 2

Supplemental Digital Content is available in the text

## 1 Introduction

Coronary artery disease (CAD) is a slowly progressive disease and the most common reason of death globally, responsible for 17.9 million deaths in 2016, representing 31% of all global death causes.^[1]^ It has been certified that inflammation plays an important role in the whole progression of CAD.^[2]^ Beneficial lifestyle and guideline-recommended treating strategies are suggested for all stable CAD (SCAD) patients to reduce morbidity and mortality.^[3]^ However, the existing medical methods could not solve this disease perfectly, even the benefit from initial percutaneous transluminal coronary intervention remains undefined.^[4]^ Thus, the therapeutic choice should be more careful and optimal.

In recent years, the herbal medicine as an alternative method has been demonstrated to treat SCAD and prevent relapse effectively, even in inflammation control.^[5–8]^ According to the theory of traditional Chinese medicine (TCM), pathogen of SCAD can be considered as stagnant Qi and blood. To deal with SCAD and the inflammation, Taoren Honghua Jian granule (THJG) was ascertained by numbers of TCM physicians in China, using *Prunus persica* and *Flos carthami* as the principal element, and *Rhizoma corydalis*, *R chuanxiong*, *Radix paeoniae rubra*, *R salviae miltiorrhizae*, *R angelicae sinensis*, *R rehmanniae*, *Pericarpium citri reticulatae viride*, *R cyperi*, and *Olibanum* as adjuvant components (details in Table 1). THJG has significant influence on inhibition of platelet and improvement of hemorheology in SD mice without triggering any side effect,^[9]^ suggesting the therapeutic potential of THJG against human SCAD. Although, this efficacy should be confirmed with more examines.

**Table 1:**
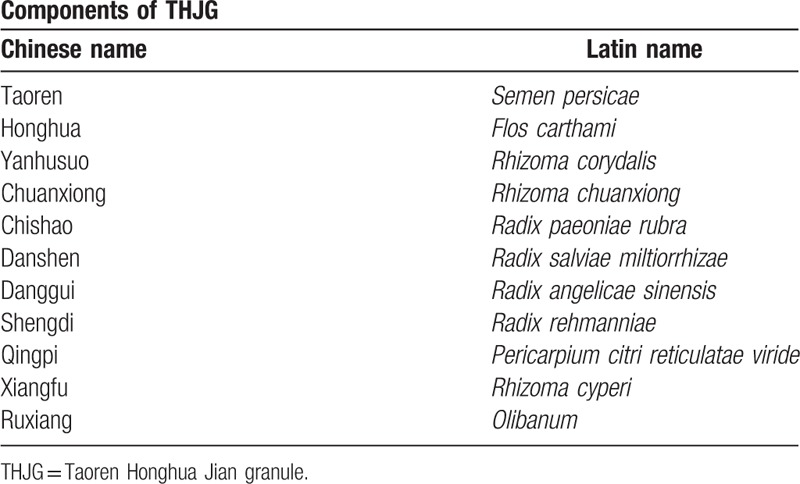
Components of THJG.

We will conduct a well-designed randomized, double-blind, placebo-controlled trial to assess the usefulness and safety of THJG on SCAD. The outcomes of this clinical research could substantiate the value of THJG to improve symptoms and inflammation of SCAD patients. To ensure the quality during the whole trial, we will use THJG, raw extraction of THJG decoction, to substitute medicinal broth. This protocol was finished in September 1, 2019 (Version 1.0).

## 2 Methods/design

### 2.1 Study design

This is a multicenter, randomized, double-blinded, placebo-controlled clinical trial with 2 parallel arms. The purpose of this research is to estimate the effectiveness and safety of THJG combined in administrate SCAD symptoms and inflammation. Altogether, we will recruit 80 patients with SCAD from 3 hospitals in China: LongHua Hospital affiliated to Shanghai University of Traditional Chinese Medicine, Shanghai Seventh People's Hospital, Gongli Hospital of Shanghai Pudong New Area, Shanggang Community Health Service Center of Shanghai Pudong New Area. Eighty participants will be randomized into THJG group or placebo group according to patients’ included sequence number equally. Patients in THJG group will take THJG (18.3 g, twice a day, orally) for continuous 4 weeks, while patients in placebo group will take THJG placebo (18.3 g, twice a day, orally) for the same duration. The Standard Protocol Items: Recommendation for Interventional Trials SPIRIT 2013 Checklist (Supplementary Material file 1).

### 2.2 Ethical issues

The trial will be planned in compliance with the Declaration of Helsinki and Ethical Guidelines for Clinical Research, and the trial protocol has been permitted by the Research Ethical Committee of LongHua Hospital affiliated to Shanghai University of Traditional Chinese Medicine (approval number: 2019LCSY008), Gongli Hospital of Shanghai Pudong New Area (approval number: 2019-023), Shanggang Community Health Service Center of Shanghai Pudong New Area (approval number: 20190610). Additionally, the protocol has been registered on Chinese Clinical Trial Registry (ChiCTR1900021772). The personal information about the potential and enrolled participants will be collected to be used only in this trial, and we will not share or maintain the personal information when unnecessary.

### 2.3 Study participants

Participants will be recruited from 3 hospitals (LongHua Hospital affiliated to Shanghai University of Traditional Chinese Medicine, Gongli Hospital of Shanghai Pudong New Area, Shanggang Community Health Service Center of Shanghai Pudong New Area). Recruitment posters and social application advertisements will be proclaimed. The objective, approaches, and potential side-effects and advantages of this research will be explicated utterly to the participants before participants signing informed consent. The planned enrollment period is 15 months.

## 3 Criteria

### 3.1 Main inclusion criteria

1. Meet the diagnostic criteria for SCAD.^[10]^
2. Up to the standard type of TCM type: stagnant Qi and blood.
3. Patients aged 18 to 70 years.
4. Patients who volunteer to participate in this trial and sign the informed consent.

### 3.2 Main exclusion criteria

1. Acute myocardial infarction and other heart disease, such as severe cardiac neurosis, diabetic cardiomyopathy, immune cardiomyopathy, etc.
2. With severe chronic or uncontrolled diseases may lose the participant.
3. Allergic constitution or allergic to THJG or has serious adverse reactions.
4. With opioid analgesic, sedative hypnotic drug, and alcohol abuse history.
5. Serious liver or kidney function damage.
6. Pregnancy, or have no plans of pregnancy, or feeding women.

## 4 Interventions

### 4.1 TCM intervention

In THJG group, patients will be instructed to dissolve THJG (18.3 g) in 200 mL hot water and take the solution orally twice a day for 4 weeks, while patients in the placebo group will take THJG placebo in the same way.

The THJG will be manufactured, packaged, and labeled by PuraPharm Corporation in Nanning (website: https://www.purapharm.com/zh-hans/). THJG is made from the following ingredients: *Semen persicae* (Taoren, 10 g), *F carthami* (Honghua 10 g), *R corydalis* (Yanhusuo, 10 g), *R chuanxiong* (Chuanxiong, 10 g), *R paeoniae rubra* (Chishao, 10 g), *R salviae miltiorrhizae* (Danshen, 15 g), *R angelicae sinensis* (Danggui, 12 g), *R rehmanniae* (Shengdi, 10 g), *P citri reticulatae viride* (Qingpi, 10 g), *R cyperi* (Xiangfu, 10 g), and *Olibanum* (Ruxiang, 3 g).

*Processing:* Extraction: all the herbs mentioned earlier are put into a water-filled container. After a 30-minute immersion, the mixture is boiled at 100°C for 1 hour. Filter out herbal sediments, and gather herbal filtrate; Concentration: concentrate the herbal filtrate; Granule: mix the concentration with dextrin. The mixture is spray dried to generate dry mash. Then mix the dry mash with magnesium stearate into powders, shattered and screened with a mesh size of 80. Finally, the THJGs are packed in individual bags (18.3 g per bag).

For the control group, THJG placebo contains 90% bitterant, edible lactose, starch, and pigment (such as lemon yellow, caramel pigment, and sunset yellow), are also made from 10% THJG to achieve the similar color, smell, taste, and texture as THJG.

At baseline (Visit 1), patients begin to receive THJG or THJG placebo and laboratory tests. At weeks 2 and 4, patients will visit again (Visits 2 and 3) and perform laboratory tests. Medicine intervention will be finished at week 4. Then at week 8, the follow-up visit (Visit 4) will be taken through a phone call. Every patient should visit and do laboratory tests within 3 days after the original time point.

### 4.2 Western medicine intervention

Besides, if necessary patients in the 2 groups could be given nitroglycerin (allowed to be taken at a dose of 0.5 g sublingual) as other common western medical treating.

### 4.3 Forbidden treatments and drugs combination

1. Any other TCM methods are not allowed (herbs except for THJG or THJG placebo, acupuncture, cupping, plaster, etc).
2. In the run-in and study period, usage of aspirin, BBs, CCBs, nitrates except for nitroglycerin and lipid-lowering drugs, in the previous 4 weeks is allowed to continue and alteration is proscribed.
3. The dosage, duration, and name of any intervention must be recorded thoroughly in the case report forms (CRFs).

### 4.4 Randomization and allocation

Shanghai Medical Clinical Research Center (CRC, website: http://www.scrcnet.org) will control the randomization and create a random number listing by SAS 9.1 PROC PLAN. This list contains 2 matching groups: 1st group is sequence number of inclusion, 2nd is the THJG or placebo group.

Pharmaceutical factory will call the CRC to acquire the random number listing after completing drugs, then attach the sequence number to boxes of medicine (THJG or placebo) according to the listing. Medicine will be transported to the CRC, administrated by a trained staff.

All the investigators (except for the CRC and pharmaceutical factory) will not know the corresponding relations between sequence numbers and different groups until the trials are completed.

When a participant is eligible to be included, the investigator will make a central telephone call and give him/her the medicine (THJG or the placebo) from the CRC administrator according to the central call.

### 4.5 Blinding and adverse event

In this trial, the investigators, physicians, nurses, laboratory personnel, statisticians (analyze outcomes), and other participants have no access to the random listing, and will not know the corresponding relations between sequence numbers and different groups until the whole trial is finished (including the statistics). All the researchers will not be in contact with the CRC and the pharmaceutical factory. Additionally, the CRC and pharmaceutical factory will be isolated from all related researchers and participants.

If an adverse event (AE) is reported, researchers should record it immediately and provide a proper emergency treatment, in case of serious adverse event, then report this to the Institutional Review Board within 24 hours. Then, the chief of Longhua Hospital ethic committee, CRC, and the highest investigators will decide to break the blinding or not after a short meeting. If an unblinding decision is made, then the intervention information of this participant, THJG, or THJG placebo will be offered by CRC.

## 5 Outcomes

### 5.1 Primary outcomes

#### 5.1.1 Integral TCM syndrome score (from baseline to week 4)

Integral TCM syndrome score is a widely used system to evaluate the efficacy of Chinese herb treating SCAD patients, which are stagnant Qi and blood type in China.^[11]^ This assessment form is constitutive of 2 parts: symptom part and sign part. In detail, there are total 8 appraisable items, angina, chest distress, palpitation, vexation, insomnia, lassitude, dizziness, and color of lips and snails, respectively. Each item consists of 4 levels and regarding scores. Wholly, the minimum score is 0 and the maximum score is 33. The higher scores of participants mean a severer condition. The scores and details of TCM syndrome are given in Tables 2 and 3.

**Table 2:**
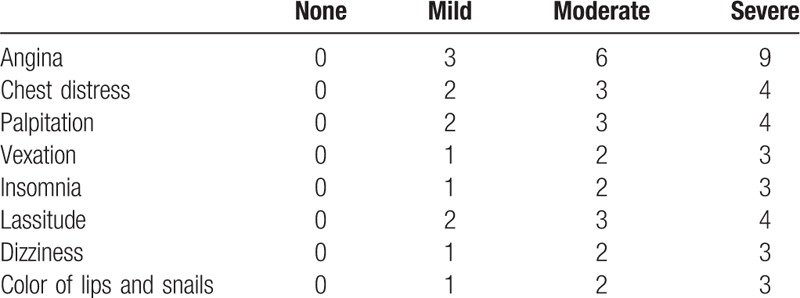
Traditional Chinese medicine symptom score.

**Table 3:**
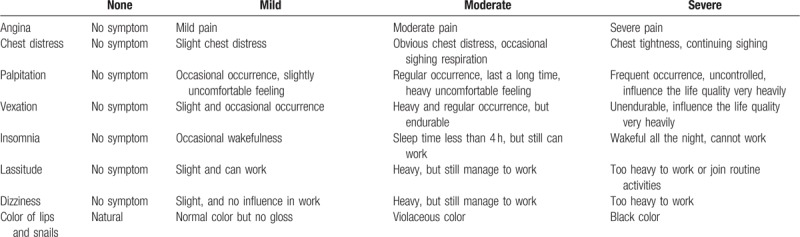
Traditional Chinese medicine symptom details.

### 5.2 Secondary outcomes

#### 5.2.1 Integral TCM syndrome score (from baseline to weeks 2 and 8)

Integral TCM syndrome score from baseline to weeks 2 and 8 will be calculated as one of the secondary outcomes.

#### 5.2.2 Inflammation indexes (from baseline to week 4)

We will monitor blood tests to certify if THJG could improve the inflammation condition, including the blood fat, *C*-reactive protein, tumor necrosis factor α, lymphocyte type, complement level, interleukin (IL, including IL-2, IL-6, IL-8, and IL-10), and immune globulin (Ig, including IgA, IgM, and IgG).

#### 5.2.3 Seattle Angina Questionnaire (SAQ) (from baseline to weeks 2, 4, and 8)

The SAQ is a disease-specific and functional questionnaire measuring 5 dimensions of health condition with 19 items: exertional capacity, anginal stability, anginal frequency, treatment satisfaction, and disease perception. Each question has 5 choices and regarding 1 to 5 scores (6 for the anginal frequency scale). Groups of studies certify the validity, responsiveness, and reliability of this evaluative instrument. Additionally, it is self-administrated and brief, requiring <5 minutes to complete. The SAQ can offer sensitive and clinically valuable outcome measures in cardiovascular research, widely used in SCAD trials.^[12–14]^

#### 5.2.4 Major adverse cardiovascular events (from baseline to weeks 2, 4, and 8)

We will calculate the rate of major adverse cardiovascular events (MACE), which comprised fatal CVDs, nonfatal MI, nonfatal cardiac arrest, nonfatal stroke, and cardiac interventions (percutaneous coronary intervention and coronary artery bypass grafting) from randomization to the end of 8 follow-up completion. This outcome measure is commonly used in clinical trials.^[15–17]^

#### 5.2.5 Athens Insomnia scale (from baseline to weeks 2, 4, and 8)

Comprehensive evidence was found that there is a link between insomnia symptoms and CAD.^[18]^ Athens Insomnia scale (AIS) is a frequently used and invaluable global insomnia symptom questionnaire in adolescents, consisting of 8 items: sleep induction, awakenings during the night, early morning awakening, total sleep duration, sleep quality during the night, emotion during the day time, functioning capacity, and sleepiness during the day time. Each item rates 0 to 3 (with 0 regarding to “no problem at all” and 3 “very serious problem”), thus maximum score is 24, which implies the most severe degree of insomnia. The higher score suggests the worse sleep quality.^[19]^

#### 5.2.6 36-Item short form health survey (from baseline to weeks 2, 4, and 8)

The 36-item short form health survey (SF-36) is a generic health status tool for measuring physical status of SCAD patients.^[20,21]^ The SF-36 consists of 36 items with 8 subscales, including physical function, role physical, bodily pain, and global health, vitality, social function, role emotional, and mental health.^[22]^

### 5.3 Safety assessments

To assure the safety of participants, the patients’ blood routine, urine routine, feces routine, kidney and liver function, brain natriuretic peptide (BNP), echocardiography, and electrocardiograph will be tested on schedule. Patients will also be asked about several of adverse effects at each visit. When an AE is claimed, researchers will record it and provide an appropriate treatment, then report them to the Institutional Review Board within 24 hours. We also will figure out the real causes of all AEs.

To protect patients’ privacy, all participants will be visited in a closed room. Participants can raise questions and withdraw at any time.

### 5.4 Participant timeline

**Figure 1:**
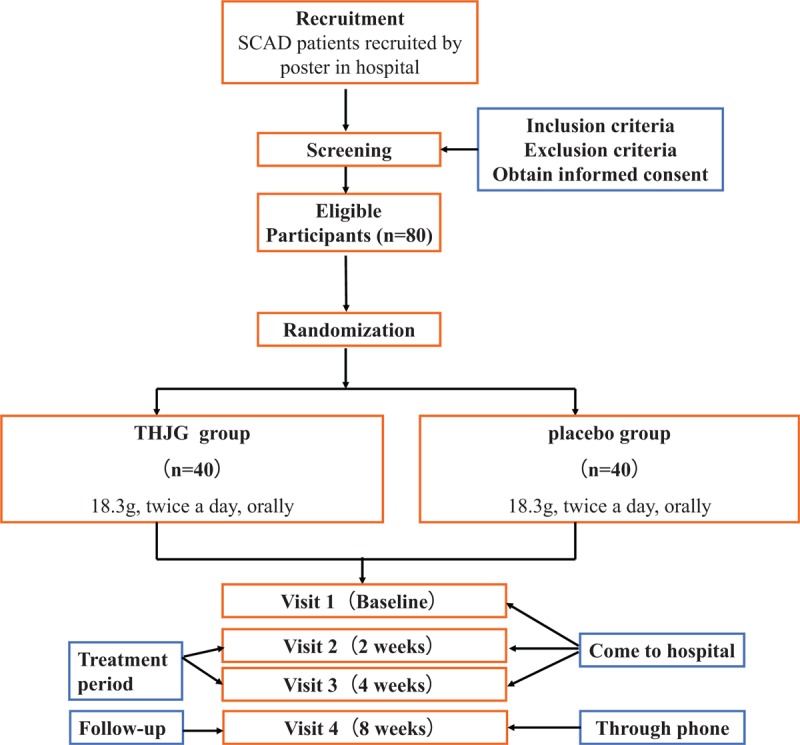
Project overview. SCAD = stable coronary artery disease, THJG = Taoren Honghua Jian granule.

Recruitment will start in October 2019 and end in January 2021. The last visit will be finished before January 31, 2021. The recruitment process is shown in Figure 1, and the schedule is given in Table 4.

**Table 4:**
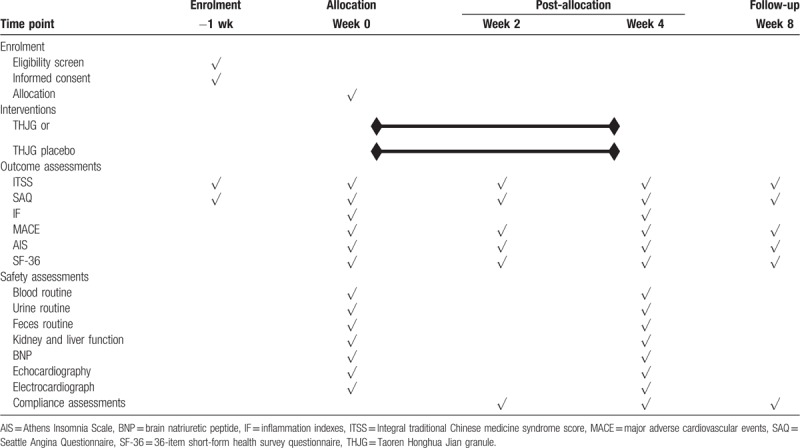
Schedule of enrollment and assessments (a separate Word file).

### 5.5 Data collection and monitoring

In this 8-weeks’ trial, all participants will be treated with THJG or THJG placebo for 4 weeks and followed-up for another 4 weeks. Five rounds (the baseline, week 2, week 4, and week 8) of disease evaluations and safety evaluations, and 2 rounds (the baseline, and week 4) blood examinations will be recorded in CRFs at each visit point, then entered in Epidata software (Version 3.1) by 2 statisticians independently every month. Difference will be solved by discussion and a 3rd party. All the trained data processors come from the CRC to keep the quality of data collection.

### 5.6 Quality control

Longhua Hospital affiliated with Shanghai University of Traditional Chinese Medicine (http://www.longhua.net/ywsy/gzlc/287.jhtml) is responsible for quality controlling and training for all investigators.

At the beginning of this trial, physicians, nurses, and laboratory personnel from 3 research centers will receive systematic training together to completely apprehend the procedure of the whole study. As the trial starts, a trained investigator will be sent to each center every week to check the following:

(1) Number of recruited participants in each center.
(2) Included SCAD patients meet the inclusion criteria and do not meet the exclusion criteria.
(3) All the participants have signed the informed consent and follow the study process.
(4) Clinicians have written the CRFs in time.

### 5.7 Sample size calculation

We conducted a pre-experiment of THJG and placebo treating SCAD from June 2018 to December 2018. According to the formula, 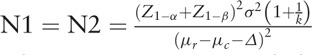, N1 and N2 are the patient numbers in THJG group and placebo group; *Z*_1–*α*_ = 1.96 when *α* = 0.05; *Z*_1–*β*_ = 1.282 when 1 − *β* = 0.90; *σ* (standard deviation of 2 groups) = 7.7; *k* = 1; *μ*_*r*_–*μ*_*c*_ (the difference of integral TCM syndrome score of THJG and control group) = 4; Δ = 0. Therefore, we plan to recruit 80 participants in total (40 of them in each group) to achieve a 90% power and a 2-sided 5% significance level in detecting the therapeutic differences between 2 groups.

### 5.8 Statistical analysis

A trained statistician from CRC will complete all the statistical analysis, by Statistical Packages of Social Sciences software (version 24.0). Intention-to-treat approach for effectiveness and safety analysis will be used. As dealing the missing values, last-observation-carried-forward method will be applied. The mean ± standard deviation will be chosen for the description of continuous variables and the percentage is chosen to describe the variable values. Continuous variables, which are kept in the normal distribution, will be analyzed by Student *t* test; or else, nonparametric tests will be adopted. All statistical tests are 2-sided and *P* < 0.05 means statistically significant.

## 6 Discussion

The treatment of SCAD has been significantly improved in recent years, especially the inflammation theory gains more attention. But to benefit patients and ameliorate the inflammation condition as many, these treatments wait to be updated. THJG consists of *R corydalis*, *S persicae*, *F carthami*, *R chuanxiong*, *R paeoniae rubra*, *R salviae miltiorrhizae*, *R angelicae sinensis*, *R rehmanniae*, *Fructus aurantii immaturus*, *R cyperi*, and *Olibanum*. Previous study proved that the main components *S persicae* and *F carthami* could alleviate inflammation and regular apoptosis, suggesting the potential beneficial effect of THJG on SCAD that should be valued by critical-designed trial.

We went through PubMed, Web of Science, Embase, the Cochrane Library, China National Knowledge Infrastructure, Wan Fang Database, VIP Journal Integration Platform, Chinese BioMedical database, and Clinical Trials up to March 2019, noticing no evidence about the efficiency and safety of THJG in treating SCAD patients. Therefore, this clinical trial will be the 1st multicenter, randomized, double-blind, placebo-controlled research that investigates the efficacy, safety, and inflammation improvement of THJG in treating SCAD.

To assure the exploration reliability, we design a 3-center, randomized, double-blind, controlled clinical trial. In addition, TCM syndrome score, a widely used for evaluation of Chinese herbs treatment, will be used to measure the symptoms improvement of the SCAD patients. Inflammation indexs, SAQ, and MACE also will be observed. Furthermore, we will evaluate the patients’ quality of life using AIS and SF-36. The safety of THJG will be evaluated according to patients’ feelings and laboratory tests, such as blood routine, feces and urine routine, kidney and liver function, BNP, echocardiography, and electrocardiograph.

The outcomes of this research will answer whether THJG is an effective drug to treat SCAD, especially to improve the inflammation condition. Its positive results will offer SCAD patients and physicians a different treatment option. Furthermore, the trial can provide proof of THJG about life quality changing, drug safety, and symptom severity relieving.

### 6.1 Trial status

Recruitment will start in October 2019 and is expected to finish in January 2021.

## Author contributions

YRW conducted this clinical trial and draft the manuscript. All authors participated in the design of the study and performed the trial. PL supervised and coordinated the clinical trial. YYZ, YYD, YY, NZ, MJM, and WTD are responsible for recruiting the participants. JW is participated in statistical design. YJR will supervise the quality of all the tests. All authors read and approved the final manuscript. PL conceived of the study and revised the manuscript critically for important intellectual content.

## Supplementary Material

Supplemental Digital Content
