# Supplementary material for: A multicenter, randomized, double-blind, placebo-controlled trial evaluating the efficacy and safety of Taoren Honghua Jian granule in patients with stable coronary artery disease
Source: Medicine (Baltimore). 2019 Nov 1;98(44):e17753. doi: 10.1097/MD.0000000000017753 (PMC6946464; doi:10.1097/MD.0000000000017753)
Supplement: Supplemental Digital Content [file medi-98-e17753-s001.pdf]

上[illegible]员会

[illegible]大学附属龙华医院医学伦理委员会批件

伦理审议批件号：上海中医药大学附属龙华医院医学伦理委员会 2019LCSY008 号

研究名称	桃仁红花煎对冠心病患者免疫功能的影响	项目批准文号	上海中医药发展办公室 [illegible]
申办单位	上海中医药大学附属龙华医院	研究方法	多中心、随机、双盲、安慰剂对照
审查类别	初次审查	审查方式	会议审查
研究单位及主要研究者	上海中医药大[illegible]<br>上海市浦东新[illegible]<br>上海上钢社区[illegible]服务中心 [illegible]		
[illegible]委员会审议成员	刘胜、李佶、张静喆、唐志鹏、许[illegible]		
[illegible]委员会地址	上海市徐汇区宛平南路 725 号，200032		
审议时间	2019 年 1 月 31 日		
审议结论	根据中华人民共和国国家药品监督管理局 2003 年颁布实施的《药物临床试验质量管理规范》、2010 年颁布实[illegible]布的《涉及人的生物医[illegible]讨论了下列有关材料：[illegible]<br>1. 研究方案（版本号[illegible]<br>2. 研究者手册（版本[illegible]<br>3. 知情同意书（版本[illegible]<br>4. 病例报告表（CRF）[illegible]<br>5. 研究病历（版本号[illegible]<br>6. 招募广告（版本号[illegible]<br>7. 主要研究者简历及[illegible]<br>8. 试验药物加工单位[illegible]<br>本伦理委员会经审[illegible]<br>上述资料未经本委[illegible]立即（24 小时内）报告[illegible]<br>如临床试验方案、[illegible]新批准；<br>请根据跟踪审查频率，提前 1 个月递交跟踪审查报告；如出现违背方案的情况，请递交违背方案报告；研究结束后 1 个月内递交研究结题报告。		
主任委员签字	刘胜（刘胜）		
跟踪审查频率	12 个月	联系电话：	021-64385700-1318
备注	纳入第一例受试者前必须向本伦理委员会递交试验药物药检报告。		

上海中医药大学附属龙华医院
医学伦理委员会（盖章）
2019 年 1 月 31 日

Medical Ethics Committee of Longhua Hospital, Shanghai University of Traditional Chinese Medicine.

A multicenter, randomized, double-blind, placebo-controlled trial evaluating the efficacy and safety of Taoren Honghua Jian granule in patients with stable coronary artery disease

Opinion of Medical Ethics of Committee of Longhua Hospital, Shanghai University of Traditional Chinese Medicine

Approval number: medical ethics committee of longhua hospital affiliated to Shanghai university of Chinese medicine 2019LCSY008

Research name

Project approval document

Sponsor

Longhua Hospital, Shanghai University of Traditional Chinese Medicine

Shanghai Development Department of Traditional Chinese Medicine ZY(2018-2020)-CCCX-2002-04

Research plan

Review type

initial

Multicenter, randomized, double-blind, placebo-controlled

Clinical research units and principal investigators

Longhua Hospital, Shanghai University of Traditional Chinese Medicine. Ping Liu
Shanghai Pudong District Gongli Hospital. Yiyue Du
Shanggang Community Health Service Center of Shanghai Pudong New Area. Ying Yang

Members of Ethics Committee

Sheng Liu,Ji Li,Jing-zhe Zhang,Zhi-peng Tang,Wen-li Xu,Wen-qin Zhou,Min-sheng Fan,Jiu-hui Li,Gang Lu,Zhi-lan Xu,Xiao-yun Chen

Address of Ethics Committee

725 South Wan-Ping Road, Shanghai 200032, China

Review date: January 31, 2019

According to the “Quality Management Standards for Drug Clinical Trials” promulgated and implemented by the State Drug Administration of the People's Republic of China in 2003, the drug clinical trial ethical review work guiding principles, and organizing committee issued the declaration of Helsinki and international medical science of the human body biological international biomedical research ethics guide moral principles in 2010. The following materials were reviewed and discussed by the ethics committee:
1.Research plan（V1.0 version,December 12,2018)
2.Researcher's manual(V1.0 version,December 12,2018)
3.Informed consent(V1.0 version,December 12,2018)
4.CRF（V1.0 version,December 12,2018)
5.Research case(V1.0 version,December 12,2018)
6.Recruitment advertisement(V1.0 version,December 12,2018)
7.Main investigator's resume and participant's resume
8.Qualification of testing drug processing unit

Our ethics committee has voted to allow you to begin this clinical trial on this date; And requirements:The above information shall not be modified without the approval of the committee; Any serious adverse events during the trial should be reported to the committee immediately (within 24 hours).

Please submit the follow-up review report 1 month in advance according to the frequency of follow-up review. If there is a breach of the programme, please submit a report of the breach; Submit the final report within 1 month after the study.

Review conclusion

Signature of the Chief

Tracking frequency：12 month

Contact number:021-64385700-1318

Note: Medicine test reports must be submitted before the first patient included

Shanghai Pudong District Gongli Hospital Ethics Committee ethics review approval

Shanghai Pudong District Gongli Hospital Ethics Committee ethics review approval

上海市浦东新区公利医院医学伦理委员会批件

Number: 2019-023

批件号：【2019】临审第(023)号

项目名称	桃仁红花煎对冠心病患者免疫功能的影[illegible]					
类别	科研项目	注册分类		试验目的	临床观察	
专业名称	中医		试验项目负责人	都乐亦	职称	主任医师
			专业负责人	都乐亦	职称	主任医师
审评材料	1. 项目评审表<br>2. 合作项目任务合同书<br>3. 龙华医院医学伦理委员会批件<br>4. 研究者手册<br>5. 研究方案<br>6. CRF 表<br>7. 知情同意书<br>8. 招募广告			主要参加单位	上海市中医药大学附属龙华医院	
	审查方式： ☐会议审查 ■快速审查					
	结论：■同意 ☐作必要的修正后同意 ☐作必要的修正后重审 ☐不同意<br>☐暂停或终止已批准的试验					
审评意见	审评意见：<br>1. 经本伦理委员会审查，同意按所批准的研究方案进行该临床研究。<br>2. 伦理委员会对该研究实施过程进行持续审查，审查频度为研究批准之日（2019 年 6 月 12 日）起：<br>☐3 个月 ☐6 个月 ■1 年<br>3. 伦理委员会有权根据研究进展情况改变持续审查频度。<br><br>主任委员（签章）：<br>日期：2019 年 6 月 12 日					

Research name: A multicenter, randomized, double-blind, placebo-controlled trial evaluating the efficacy and safety of Taoren Honghua Jian granule in patients with stable coronary artery disease

Type: Research project

Aim: Clinical obsevation

Project leader: Yiyue Du

Title: Chief physician

Subject name: Tradional Chinese Medicine

Subject leader: Yiyue Du

Main study unit: Shanghai University of Traditional Chinese Medicine Longhua Hospital

Assessment documents

1. Project review form
2. Cooperation contract
3. Medical Ethics Committee opinion of Shanghai University of Traditional Chinese Medicine Longhua Hospital
4. Researcher's manual
5. Research plan
6. CRF
7. Informed consent
8. Recruitment advertisement

Assess method: quick assessment

Conclusion: approve

Assess advice:
1. According to our assessment, we approve this clinical research.
2. We will assess this project one year later.
3. We have the right to change reassess time.

上海市浦东新区公利医院医学伦理委员会

伦理委员会地址：上海市浦东新区苗圃路 219 号 邮编：200135 联系电话：021-58858730

备注：

1、本批件将在各中心机构及其伦理审查委员会备案。如果对方案在贵机构的可行性（包括研究者的资格与经验、设备与条件等）有不同意见，请及时与本伦理审查委员会联系。

2、已批准项目须遵循本伦理审查委员会批准的方案执行，须符合 SFDA/GCP 和《赫尔辛基宣言》的原则。

3、 暂停/提前终止临床研究，请及时通知本伦理审查委员会。

4、发生严重不良事件及影响研究风险收益比的非预期事件，须及时报告本伦理审查委员会。

5、对已批准的临床研究方案、知情同意书等材料的任何修改及主要研究者更换等，须及时通知本伦理审查委员会重新审查，获得批准后执行。

6、发现违反方案情况须及时汇报。

7、根据伦理审查委员会对持续审查频度的意见，无论试验开始与否，请在持续审查日到期前 1 个月提出持续审查的申请。

8、完成临床研究，须提交结题报告供伦理审查委员会审查。

9、本批件的有效期为 1 年，逾期未实施的则自动废止。

Shanggang Community Health Service Center of Shanghai Pudong New Area
Ethics review of “A multicenter, randomized, double-blind, placebo-controlled trial evaluating the efficacy and safety of Taoren Honghua Jian granule in patients with stable coronary artery disease"

上海市浦东新区上钢社区卫生服务中心

关于"桃仁红花煎对冠心病患者免疫功能的影响"的伦理审查意见

Clinical research project：A multicenter, randomized, double-blind, placebo-controlled trial evaluating the efficacy and safety of Taoren Honghua Jian granule in patients with stable coronary artery disease

临床研究项目：桃仁红花煎对冠心病患者免疫功能的影响

Sponsor
申办者：上海中医药大学附属龙华医院
Longhua Hospital, Shanghai University of Traditional Chinese Medicine

主要研究者：刘萍
Principal investigator：Ping Liu

Sub-center
分中心单位：上海市浦东新区上钢社区卫生服务中心
Shanggang Community Health Service Center of Shanghai Pudong New Area

主要负责人：丁燕
Principal ileader：**Yan Ding**

审查日期：2019.06.10
Review time: 2019.06.10

审查类型：初始审查
Review type：Initial review

审查方式：快速审查
Review method：Quick review

审查文件：
Review file

1.研究方案：V2.0 版本，2019 年 05 月 15 日
2.研究者手册：V1.0 版本，2018 年 12 月 12 日
3.知情同意书：V1.0 版本，2018 年 12 月 12 日
4.病例报告表（CRF）：V1.0 版本，2018 年 12 月 1[illegible]
5.龙华医院伦理批件

1.Research plan: V2.0 version,version date: 2019.05.15
2. Researcher's manual(V1.0 version,December 12,2018)
3.Informed consent(V1.0 version,December 12,2018)
4.CRF（V1.0 version,December 12,2018)
5. Medical Ethics Committee opinion of Shanghai University of Traditional Chinese Medicine Longhua Hospital

审查意见：

根据我国国家食品药品监督管理局《药物临床试验伦理审查工作指导原则》（2010 年）、国家中医药管理局《中医药临床研究伦理审查管理规范》（2010 年）、卫生部《涉及人的生物医学研究伦理审查办法（试行）》（2007 年）、国家食品药品监督管理局《医疗器械临床试验规定》（2004 年）和《药物临床试验质量管理规范》（2003 年）的伦理原则，经本中心相关部门审查决定：同意。

上海市浦东新区上钢社区卫生服务中心（盖章）

According to the guidelines for the ethical review of drug clinical trials issued by the state food and drug administration of China(in 2010)、Standards for the ethical review and management of TCM clinical research issued by the state administration of traditional Chinese medicine(in 2010)、Methods for ethical review of biomedical research involving humans (trial)issued by The ministry of health(in 2007)、Clinical trial regulations for medical devices issued by the state food and drug administration（in 2004)and ethical principles of the code for the quality management of drug clinical trials,Review the decision:agreement

Shanggang Community Health Service Center of Shanghai Pudong New Area

附件 4

The 2018-2020 Three-year Action Plan for Traditional Chinese Medicine Further Development in Shanghai

上海市进一步加快中医药事业发展三年行动计划（2018 年-2020 年）

Project construction task book

项 目 建 设 任 务 书

Project number: ZY(2018-2020)-CCCX-2002-04

项目编号 ZY(2018-2020)-CCCX-2002-04

Project type: Construction of Chinese Medicine Heritage Innovation Platform

项目类别 中医药传承创新平台建设

Project name: Longhua Hospital National Traditional Chinese Medicine Heritage Innovation Project Connotation Construction

项目名称 龙华医院国家中医药传承创新工程内涵建设

Project leader: Ping Liu

项目负责人 刘萍

Project undertaker: Longhua Hospital, Shanghai University of Traditional Chinese Medicine

项目承担单位 上海中医药大学附属龙华医院（盖章）

Time: from September 2018 to December 2020

项目起止年月 2018 年 9 月 至 2020 年 12 月

Shanghai Health and Family Planning Commission
Shanghai Chinese Medicine Development Office

上海市卫生和计划生育委员会

上海市中医药发展办公室

二〇一八年制

一、项目基本信息

Project name: Intervention study on the immune injury of coronary atherosclerotic heart disease based on the theory of "heart and blood"

项目名称	基于"心主血脉"理论观察桃仁红花煎对冠状动脉粥样硬化性心脏病免疫损伤的干预研究		
项目建设单位信息 Project construction unit information			
单位名称	上海中医药大学附属龙华医院 Unit name: Longhua Hospital, Shanghai University of Traditional Chinese Medicine	单位性质	事业单位 unit type: public
通讯地址	上海市徐汇区宛平南路725号 Address: 725 South Wan-Ping Road, Xuhui District, Shanghai	邮政编码	200032 code: 200032
单位内主管部门	科技处 Administration: Technology Department	主管部门联系人	李佳 Administration leader: Ji Li
联系电话	021-64385700-6314 Contact number: 021-64385700-6314	电子信箱	lhtcmkyc@sina.com Email: lhtcmkyc@sina.com
项目负责人信息 Information of project leader			
姓名	刘萍 Name: Ping Liu	性别	女 Gender: Female
出生日期	1966.02.07 Birth: 1966.02.07	职称	主任医师 Title: Chief physician
最高学位	博士 Highest degree: Ph.D	从事专业	中医药抗动脉粥样硬化研[illegible] Major subject: Chinese medicine anti-atherosclerosis research
固定电话	64385700-3522 Phone number	移动电话	[illegible]7763029 Mobile phone number
传真号码	64398310 Fax number	电子信箱	liuping0207@126.com Email
项目经费来源（万元）			
总经费 Total funding	83.03946		
市级财政投入 Municipal financial input	59.03946		
区级财政投入	/		
单位自筹 Unit self-raising	24		
其他	/		
经费备注	/		

备注：项目建设经费采取多元投入的方式，以市级财政补助经费为主导。市级项目单位自筹经费原则上不低于30%。区属公立医疗卫生机构申报的，区财政配套经费原则上不低于20%，项目单位自筹经费原则上不低于30%。具体匹配资金额度以市财政专项评审核定金额为准。

十一、项目协议

项目建设单位

本单位承诺按照项目建设要求，根据经费预算所填写单位匹配部分，实时到账，并与市财政资助项目经费统筹管理，实物和人员经费不作为匹配经费额度。同时加强项目建设与管理，在人、财、物等方面保障项目顺利按计划完成，承担未按时完成项目的责任。

Project construction unit

The unit promises to fill in the matching parts according to the budget of the project according to the project construction requirements, and arrive at the account in real time, and coordinate with the municipal financial support project funds, and the physical and personnel funds are not used as matching funds. At the same time, the project construction and management will be strengthened, and the project will be completed in accordance with the plan in terms of people, finances and materials, and the responsibility for completing the project on time will be undertaken.

单位盖章:

年　　月　　日

项目责任单位上级主管单位（区属单位填写）

Project responsible unit

同意按要求匹配相关经费。并加强监管，确保项目顺利完成。

Agree to match the relevant funds as required. And strengthen supervision to ensure the successful completion of the project.

单位盖章:

年　　月　　日

上海市中医药发展办公室

Shanghai Chinese Medicine Development Office

同意该项目列为上海市进一步加快中医药事业发展三年行动计划（2018 年-2020 年）建设项目。

It is agreed that the project will be listed as a construction project for Shanghai to further accelerate the three-year action plan for the development of Chinese medicine (2018-2020).

年　　月　　日

密级：

Shanghai Science and Technology Commission

上海市科学技术委员会

Funded project proposal

科研计划项目(课题)任务书

Version 1.0

（V1.0版）

Project Number: 18401900200

项目（课题）编号 18401900200

Project name: Therapeutic effect of Tiaoxin Recipe on coronary heart disease with angina pectoris and anxiety and depression

项目（课题）名称 调心方治疗冠心病心绞痛伴焦虑抑郁状态的疗效研究

Start date

开始日期 2018-09-30

Finish date

结束日期 2021-09-30

Undertake unit: Shanghai University of Traditional Chinese Medicine

项目（课题）承担单位 上海中医药大学附属龙华医院（盖章）

Address: 725 South Wan-Ping Road, Xuhui District, Shanghai

通讯地址 上海市宛平南路725号

Phone: 021-64385700 Code: 200032

联系电话 021-64385700 邮政编码 200032

项目（课题）责任人 刘萍

Leader: Ping Liu

手 机 18917763029 电子邮件 liuping0207@126.com

Phone number: 18917763029

Email: liuping0207@126.com

2018年10月23日订

□卫生行业专项
■联合攻关项目
□青年科技项目

Pudong New Area Health and Family Planning Commission

浦东新区卫生和计划生育委员会

Funded project proposal

卫生计生科研项目计划任务合同书

Project name: Clinical Study on the Treatment of "Double Heart" Abnormality of Coronary Heart Disease by "Heart and Liver Treatment"

项目名称：“心肝同治”法治疗冠心病“双心”异常的临床研究

Leader: Ping Liu

负 责 人：刘萍

Undertake unit: Shanghai University of Traditional Chinese Medicine

承担单位：上海中医药大学附属龙华医院（盖章）

Phone number: 021-64385700-3623 OR 18917763029

电话/手机：02164385700-3623/18917763029

Project Number: PW2018D-11

项目编号：PW2018D-11

Duration: From December 1,2018 to November 30, 2021

起止时间：2018 年 12 月 1 日至 2021 年 11 月 30 日

Shanghai Pudong New Area Health and Family Planning Commission

上海市浦东新区卫生和计划生育委员会
二〇一八年版

培力（南寧）藥業有限公司檢測中心 **PuraPharm (Nanning) Pharmaceuticals Co.,Ltd. Testing Laboratory**
中國廣西南寧市高新技術開發區科園大道 46 號　No.46, Ke Yuan Road,Nanning New & High-tech Industrial Development Zone, Guangxi,China.

對“桃仁紅花煎顆粒”之樣品之分析報告
Report on the submitted sample identified by the client - Tao Ren Hong Hua Jian

樣品名稱 Product Description	桃仁紅花煎顆粒 Tao Ren Hong Hua Jian
樣品編號 Product Code	3350
樣品規格 Product Specification	18.3g/袋 pack
批號 Batch No.	A190053710
有效期至(年/月/日)Expiry Date（yyyy/mm/dd）	2022/03/27
本批數量 Quantity Produced	2643 袋 packs
樣品收到時狀態 Sample Receiving Condition	室溫下存放於密封鋁塑複合膜原來包裝中<br>In sealed lamination film package under ambient condition.
製造商 Manufacturer	生產部 Production Department
委託檢驗單位 Inspected Entity	生產部 Production Department
來源地 Region of Origin	生產部 Production Department
目的地 Region of Destination	培力（南寧）藥業有限公司檢測中心<br>PuraPharm (Nanning) Pharmaceuticals Co.,Ltd. Testing Laboratory
檢驗日期(年/月/日)Testing Period（yyyy/mm/dd）	2019/04/16 - 2019/04/21

測試項目、分析方法及分析結果 Test Requested, Test Method and Test Results
請參考續頁。Please refer to the following page(s)

****

培力（南寧）藥業有限公司檢測中心代表簽名
Signed for and on behalf of PuraPharm (Nanning) Pharmaceuticals Co.,Ltd. Testing Laboratory

培力（南寧）藥業有限公司檢測中心 PuraPharm (Nanning) Pharmaceuticals Co.,Ltd. Testing Laboratory
郵編(Postal No.)：530007　　電話(Tel)：0771-3218026　　傳真(Fax)：0771-3216602

測試項目及分析方法 Test Requested and Test Method

測試項目 Test Items	參考方法 Reference Method
1. 性狀 Appearance	中國藥典, 2015 年版, 第四部, 通則 0104<br>The Pharmacopoeia of the People's Republic of China 2015, Vol.4, General chapter 0104
2. 水分 Determination of Water	中國藥典, 2015 年版, 第四部, 通則 0832<br>The Pharmacopoeia of the People's Republic of China 2015, Vol.4, General chapter 0832
3. 粒度 Size of Granule	中國藥典, 2015 年版, 第四部, 通則 0982<br>The Pharmacopoeia of the People's Republic of China 2015, Vol.4, General chapter 0982
4. 溶化性<br>Determination of Dispersibility	中國藥典, 2015 年版, 第四部, 通則 0104<br>The Pharmacopoeia of the People's Republic of China 2015, Vol.4, General chapter 0104
5. 裝量差異 Variation in content	中國藥典, 2015 年版, 第四部, 通則 0104<br>The Pharmacopoeia of the People's Republic of China 2015, Vol.4, General chapter 0104
6. 重金屬及有害元素<br>Heavy Metals and Toxic Elements	中國藥典, 2015 年版, 第四部, 通則 2321；電感耦合等離子體質譜法；<br>The Pharmacopoeia of the People's Republic of China 2015, Vol.4, General chapter 2321；ICP-MS
7. 農藥殘留 Pesticides Residues	香港中醫藥管理委員會《中成藥註冊申請手冊》<br>Chinese Medicine Council of Hong Kong《Application Form: Registration of proprietary Chinese medicines》
8. 微生物限度 Microbial Limit<br>- 需氧菌總數 TAMC (Total Aerobic Microbial Count)<br>- 霉菌和酵母菌總數 TYMC (Total Yeast and Mold Microbial Count)<br>- 大腸埃希菌 Escherichia Coli	中國藥典, 2015 年版, 第四部, 通則 1105、1106、1107<br>The Pharmacopoeia of the People's Republic of China 2015, Vol.4, General chapter 1105，1106，1107

****

培力（南寧）藥業有限公司檢測中心 PuraPharm (Nanning) Pharmaceuticals Co.,Ltd. Testing Laboratory
郵編(Postal No.)：530007 電話(Tel)：0771-3218026 傳真(Fax)：0771-3216602

分析結果 Test Results

測試項目 Test Items	品質標準 Quality Specification	桃仁紅花煎顆粒<br>Tao Ren Hong Hua Jian
1. 性狀 Appearance	顆粒均勻，色澤一致，無吸潮、結塊、潮解等現象。<br>Size and colour of granules are uniform, There should be no moisture absorption, clumping, deliquescence, etc.	符合規定 Conform
2. 水分 Determination of Water	≤6.5% (W/W)	3.2%<br>符合規定 Conform
3. 粒度 Size of Granule	不能通過一號篩和能通過五號篩的顆粒和粉末總和不得超過 15% Sum of weight of granules that cannot pass through sieve No.1 and weight of powder that can pass through sieve No.5≤15%	2%<br>符合規定 Conform
4. 溶化性<br>Determination of Dispersibility	應符合規定 Shall meet the requirement	符合規定 Conform
5. 裝量差異 Variation in content	應符合規定 Shall meet the requirement	符合規定 Conform
6. 重金屬及有害元素#<br>Heavy Metals and Toxic Elements		
- 銅 Copper (Cu)	不得過 150.00mg/kg<br>Cu≤150.00 mg/kg	1.227mg/kg<br>符合規定 Conform
- 砷 Arsenic (As)	不得過 41.67mg/kg 或 1500μg/日<br>As≤41.67mg/kg or 1,500μg/day	0.335mg/kg（12.06μg/日 day）<br>符合規定 Conform
- 鎘 Cadmium (Cd)	不得過 97.22mg/kg 或 3500μg/劑<br>Cd≤97.22mg/kg or 3,500μg/dose	0.024mg/kg（0.86μg/劑 dose）<br>符合規定 Conform
- 鉛 Lead (Pb)	不得過 4.97mg/kg 或 179μg/日<br>Pb≤4.97mg/kg or 179μg/day	0.073mg/kg（2.63μg/日 day）<br>符合規定 Conform
- 汞 Mercury (Hg)	不得過 1.00mg/kg 或 36μg/日<br>Hg≤1.00mg/kg or 36μg/day	＜0.001mg/kg（＜0.04μg/日 day）<br>符合規定 Conform
7. 農藥殘留#<br>Pesticides Residues		
- 艾氏劑及狄氏劑<br>Aldrin & Dieldrin	艾氏劑及狄氏劑兩者之和≤0.05 毫克/千克<br>Sum of aldrin & dieldrin≤0.05 mg/kg<br>（檢出限艾氏劑 1.77μg/kg,狄氏劑 2.43μg/kg<br>LOD aldrin 1.77μg/kg, dieldrin 2.43μg/kg）	未檢出 Not detected<br>符合規定 Conform
- 氯丹 Chlordane	順式-,反式及氧化氯丹之和≤0.05 毫克/千克<br>Sum of cis-,trans- & oxy-chlordane≤0.05 mg/kg<br>（檢出限順式氯丹 2.05μg/kg, 反式氯丹 1.81μg/kg, 氧化氯丹 2.21μg/kg<br>LOD cis- chlordane 2.05μg/kg, trans- chlordane 1.81μg/kg, oxy-chlordane 2.21μg/kg）	未檢出 Not detected<br>符合規定 Conform
- 滴滴涕 DDT	4,4'-滴滴涕, 2,4'-滴滴涕, 4,4'-滴滴伊及 4,4'-滴滴滴之和≤1.0 毫克/千克<br>Sum of *p, p*'-DDT, *o,p*'-DDT, *p,p*'-DDE, *p,p*'-TDE ≤1.0 mg/kg<br>（檢出限 4,4'-滴滴涕 3.73μg/kg, 2,4'-滴滴涕 4.48μg/kg, 4,4'-滴滴伊 2.29μg/kg, 4,4'-滴滴滴 2.88μg/kg<br>LOD *p, p*'-DDT 3.73μg/kg, *o,p*'-DDT 4.48μg/kg, *p,p*'-DDE 2.29μg/kg, *p,p*'-TDE 2.88μg/kg）	未檢出 Not detected<br>符合規定 Conform
- 異狄氏劑 Endrin	≤0.05 mg/kg<br>（檢出限 LOD 3.78μg/kg）	未檢出 Not detected<br>符合規定 Conform

****

編制 Report compiled by： [signature] 審核 Reviewed by： [signature]

培力（南寧）藥業有限公司檢測中心 PuraPharm (Nanning) Pharmaceuticals Co.,Ltd. Testing Laboratory
郵編(Postal No.)：530007 電話(Tel)：0771-3218026 傳真(Fax)：0771-3216602

- 七氯 Heptachlor	七氯及環氧七氯之和≤0.05 毫克/千克<br>Sum of heptachlor & heptachlor epoxide≤ 0.05 mg/kg<br>（檢出限七氯 1.78μg/kg，環氧七氯 1.97μg/kg<br>LOD heptachlor 1.78μg/kg, heptachlor epoxide 1.97μg/kg）	未檢出 Not detected<br>符合規定 Conform
- 六氯苯 Hexachlorobenzene	≤0.1 mg/kg<br>（檢出限 LOD 2.16μg/kg）	未檢出 Not detected<br>符合規定 Conform
- 六六六 Hexachlorocyclohexane	α-,β-及 δ-異構體之和≤0.3 毫克/千克<br>Sum of α-,β-,δ- isomers≤0.3 mg/kg<br>（檢出限 α-六六六 1.61μg/kg, β-六六六 2.96μg/kg, δ-六六六 1.43μg/kg<br>LOD α- HCH 1.61μg/kg, β- HCH 2.96μg/kg, δ- HCH 1.43μg/kg）	3.02×10^{-3}mg/kg<br>符合規定 Conform
- 林丹 Lindane	≤0.6 mg/kg<br>（檢出限 LOD 1.56μg/kg）	未檢出 Not detected<br>符合規定 Conform
- 五氯硝基苯 Quintozene	五氯硝基苯，五氯苯胺及甲基五氯硫基苯之和≤1.0 毫克/千克<br>Sum of quintozene, pentachloroaniline and methyl pentachlorophenyl sulphide≤1.0 mg/kg<br>（檢出限五氯硝基苯 1.92μg/kg，五氯苯胺 1.59μg/kg，甲基五氯硫基苯 1.33μg/kg<br>LOD quintozene 1.92μg/kg , pentachloroaniline 1.59μg/kg , methyl pentachlorophenyl sulphide 1.33μg/kg）	未檢出 Not detected<br>符合規定 Conform
8. 微生物限度#<br>Microbial Limit		
-需氧菌總數 TAMC (Total Aerobic Microbial Count)	≤500cfu/g	＜10cfu/g<br>符合規定 Conform
-霉菌和酵母菌總數 TYMC (Total Yeast and Mold Microbial Count)	≤100cfu/g	＜10cfu/g<br>符合規定 Conform
- 大腸埃希菌 Escherichia Coli	不得檢出/克 Not detected/g	未檢出/克 Not detected/g<br>符合規定 Conform

****

編制 Report compiled by：　　審核 Reviewed by：

培力（南寧）藥業有限公司檢測中心 PuraPharm (Nanning) Pharmaceuticals Co.,Ltd. Testing Laboratory

郵編(Postal No.)：530007　電話(Tel)：0771-3218026　傳真(Fax)：0771-3216602

註：Remarks

1、 報告無"檢驗/檢測報告專用章"和缺騎縫章無效。

Test report without the stamp of "For Test/Test Report Only" is not authorized.

2、 複製的報告未重新加蓋"檢驗/檢測報告專用章"及騎縫章無效。

Certified copy of test reportwithout the stamp of"For Test/Test Report Only" is not authorized.

3、 報告無編制、審核及本中心代表簽字無效。

Test report must be authorized by the signature of the bodies of preparation, verification and representative of the testing centre.

4、 報告塗改、缺頁無效。

Test report with modification or lacking of page(s) is not authorized.

5、 對報告若有異議，請於收到報告之日起十五日內向檢驗單位提出書面申訴，否則按認可檢驗報告處理。本中心異議受理電話 0771-3218026。

The client is aggrieved by the test result. A request for review shall state in writing the reasons relied upon and shall be made to the testing centre within 15 days after receipt of the authorized test report. No requirement will be accepted after the definite time. Inquiry hot line is 0771-3218026.

6、 送樣委託檢驗，樣品名稱為委託單位自報名稱，報告僅對來樣負責。部份複製檢驗/檢測報告無效。

Name of tested product is provided by the client. The report will refer only to the sample tested. Copy of partial of the test report is not authorized.

# - 其他微生物限度要求: Other requirements for Microbial Limit Test

1. 含動物類藥材（包括提取物）的中藥固體製劑，每 10g 不得檢出沙門菌。

TCM solid preparation containing animal raw materials (including extract): Absence of Salmonella (10g).

2. 含藥材原粉的中藥固體製劑，每 10g 不得檢出沙門菌；耐膽鹽革蘭陰性菌應小於 10^2cfu（1g）。

TCM solid preparation containing herbal raw powders: Absence of Salmonella (10g); Not more than 10^2cfu of Bile-tolerant gram-negative bacteria (1g).

*** End of Report 報告完 ***

培力（南寧）藥業有限公司檢測中心 **PuraPharm (Nanning) Pharmaceuticals Co.,Ltd. Testing Laboratory**

中國廣西南寧市高新技術開發區 科園大道 46 號

No.46, Ke Yuan Road,Nanning New & High-tech Industrial Development Zone, Guangxi,China.

對"桃仁紅花煎顆粒安慰劑"之樣品之分析報告

Report on the submitted sample identified by the client - Tao Ren Hong Hua Jian Ke Li An Wei Ji

樣品名稱 Product Description	桃仁紅花煎顆粒安慰劑<br>Tao Ren Hong Hua Jian Ke Li An Wei Ji
樣品編號 Product Code	3351
樣品規格 Product Specification	18.3g/袋 pack
批號 Batch No.	A190053710.
有效期至(年/月/日)Expiry Date（yyyy/mm/dd）	2022/03/27
本批數量 Quantity Produced	2693 袋 packs
樣品收到時狀態 Sample Receiving Condition	室溫下存放於密封鋁塑複合膜原來包裝中<br>In sealed lamination film package under ambient condition.
製造商 Manufacturer	生產部 Production Department
委託檢驗單位 Inspected Entity	生產部 Production Department
來源地 Region of Origin	生產部 Production Department
目的地 Region of Destination	培力（南寧）藥業有限公司檢測中心<br>PuraPharm (Nanning) Pharmaceuticals Co.,Ltd. Testing Laboratory
檢驗日期(年/月/日)Testing Period（yyyy/mm/dd）	2019/04/16 - 2019/04/21

測試項目、分析方法及分析結果 Test Requested, Test Method and Test Results

請參考續頁。Please refer to the following page(s)

****

培力（南寧）藥業有限公司檢測中心代表簽名

Signed for and on behalf of PuraPharm (Nanning) Pharmaceuticals Co.,Ltd. Testing Laboratory

測試項目及分析方法 Test Requested and Test Method

測試項目 Test Items	参考方法 Reference Method
1. 性狀 Appearance	中國藥典, 2015 年版, 第四部, 通則 0104<br>The Pharmacopoeia of the People's Republic of China 2015, Vol.4, General chapter 0104
2. 水分 Determination of Water	中國藥典, 2015 年版, 第四部, 通則 0832<br>The Pharmacopoeia of the People's Republic of China 2015, Vol.4, General chapter 0832
3. 粒度 Size of Granule	中國藥典、2015 年版、第四部、通則 0982<br>The Pharmacopoeia of the People's Republic of China 2015, Vol.4, General chapter 0982
4. 溶化性<br>Determination of Dispersibility	中國藥典, 2015 年版, 第四部, 通則 0104<br>The Pharmacopoeia of the People's Republic of China 2015, Vol.4, General chapter 0104
5. 裝量差異 Variation in content	中國藥典, 2015 年版, 第四部, 通則 0104<br>The Pharmacopoeia of the People's Republic of China 2015, Vol.4, General chapter 0104
6. 重金屬及有害元素<br>Heavy Metals and Toxic Elements	中國藥典, 2015 年版, 第四部, 通則 2321；電感耦合等離子體質譜法；<br>The Pharmacopoeia of the People's Republic of China 2015, Vol.4, General chapter 2321；ICP-MS
7. 農藥殘留 Pesticides Residues	香港中醫藥管理委員會《中成藥註冊申請手冊》<br>Chinese Medicine Council of Hong Kong《Application Form: Registration of proprietary Chinese medicines》
8. 微生物限度 Microbial Limit	中國藥典, 2015 年版, 第四部, 通則 1105、1106、1107<br>The Pharmacopoeia of the People's Republic of China 2015, Vol.4, General chapter 1105，1106，1107
- 需氧菌總數 TAMC (Total Aerobic Microbial Count)	
- 霉菌和酵母菌總數 TYMC (Total Yeast and Mold Microbial Count)	
- 大腸埃希菌 Escherichia Coli	

****

培力（南寧）藥業有限公司檢測中心 PuraPharm (Nanning) Pharmaceuticals Co.,Ltd. Testing Laboratory
郵編(Postal No.)：530007 電話(Tel)：0771-3218026 傳真(Fax)：0771-3216602

分析結果 Test Results

測試項目 Test Items	品質標準 Quality Specification	桃仁紅花煎顆粒安慰劑 Tao Ren Hong Hua Jian Ke Li An Wei Ji
1. 性狀 Appearance	顆粒均勻，色澤一致，無吸潮、結塊、潮解等現象。Size and colour of granules are uniform, There should be no moisture absorption, clumping, deliquescence, etc.	符合規定 Conform
2. 水分 Determination of Water	≤6.5% (W/W)	3.4%<br>符合規定 Conform
3. 粒度 Size of Granule	不能通過一號篩和能通過五號篩的顆粒和粉末總和不得超過 15% Sum of weight of granules that cannot pass through sieve No.1 and weight of powder that can pass through sieve No.5≤15%	8%<br>符合規定 Conform
4. 溶化性 Determination of Dispersibility	應符合規定 Shall meet the requirement	符合規定 Conform
5. 裝量差異 Variation in content	應符合規定 Shall meet the requirement	符合規定 Conform
6. 重金屬及有害元素# Heavy Metals and Toxic Elements		
- 銅 Copper (Cu)	不得過 150.00mg/kg<br>Cu≤150.00 mg/kg	0.130mg/kg<br>符合規定 Conform
- 砷 Arsenic (As)	不得過 41.67mg/kg 或 1500μg/日<br>As≤41.67mg/kg or 1,500μg/day	0.034mg/kg（1.22μg/日 day）<br>符合規定 Conform
- 鎘 Cadmium (Cd)	不得過 97.22mg/kg 或 3500μg/劑<br>Cd≤97.22mg/kg or 3,500μg/dose	0.002mg/kg（0.07μg/劑 dose）<br>符合規定 Conform
- 鉛 Lead (Pb)	不得過 4.97mg/kg 或 179μg/日<br>Pb≤4.97mg/kg or 179μg/day	0.014mg/kg（0.50μg/日 day）<br>符合規定 Conform
- 汞 Mercury (Hg)	不得過 1.00mg/kg 或 36μg/日<br>Hg≤1.00mg/kg or 36μg/day	<0.001mg/kg（<0.04μg/日 day）<br>符合規定 Conform
7. 農藥殘留# Pesticides Residues		
- 艾氏劑及狄氏劑 Aldrin & Dieldrin	艾氏劑及狄氏劑兩者之和≤0.05 毫克/千克<br>Sum of aldrin & dieldrin≤0.05 mg/kg<br>（檢出限艾氏劑 1.77μg/kg,狄氏劑 2.43μg/kg<br>LOD aldrin 1.77μg/kg, dieldrin 2.43μg/kg）	未檢出 Not detected<br>符合規定 Conform
- 氯丹 Chlordane	順式-,反式及氧化氯丹之和≤0.05 毫克/千克<br>Sum of cis-,trans- & oxy-chlordane≤0.05 mg/kg<br>（檢出限順式氯丹 2.05μg/kg, 反式氯丹 1.81μg/kg, 氧化氯丹 2.21μg/kg<br>LOD cis- chlordane 2.05μg/kg, trans- chlordane 1.81μg/kg, oxy-chlordane 2.21μg/kg）	未檢出 Not detected<br>符合規定 Conform
- 滴滴涕 DDT	4,4'-滴滴涕, 2,4'-滴滴涕, 4,4'-滴滴伊及 4,4'-滴滴滴之和≤1.0 毫克/千克<br>Sum of *p, p'*-DDT, *o,p'*-DDT, *p,p'*-DDE, *p,p'*-TDE ≤1.0 mg/kg<br>（檢出限 4,4'-滴滴涕 3.73μg/kg, 2,4'-滴滴涕 4.48μg/kg, 4,4'-滴滴伊 2.29μg/kg, 4,4'-滴滴滴 2.88μg/kg<br>LOD *p, p'*-DDT 3.73μg/kg, *o,p'*-DDT 4.48μg/kg, *p,p'*-DDE 2.29μg/kg, *p,p'*-TDE 2.88μg/kg）	未檢出 Not detected<br>符合規定 Conform
- 異狄氏劑 Endrin	≤0.05 mg/kg<br>（檢出限 LOD 3.78μg/kg）	未檢出 Not detected<br>符合規定 Conform

****

編制 Report compiled by：　　　　審核 Reviewed by：

培力（南寧）藥業有限公司檢測中心 PuraPharm (Nanning) Pharmaceuticals Co.,Ltd. Testing Laboratory
郵編(Postal No.)：530007　電話(Tel)：0771-3218026　傳真(Fax)：0771-3216602

- 七氯 Heptachlor	七氯及環氧七氯之和≤0.05 毫克/千克<br>Sum of heptachlor & heptachlor epoxide≤ 0.05 mg/kg<br>（檢出限七氯 1.78μg/kg, 環氧七氯 1.97μg/kg<br>LOD heptachlor 1.78μg/kg, heptachlor epoxide 1.97μg/kg）	未檢出 Not detected<br>符合規定 Conform
- 六氯苯 Hexachlorobenzene	≤0.1 mg/kg<br>（檢出限 LOD 2.16μg/kg）	未檢出 Not detected<br>符合規定 Conform
- 六六六 Hexachlorocyclohexane	α-,β-及 δ-異構體之和≤0.3 毫克/千克<br>Sum of α-,β-,δ- isomers≤0.3 mg/kg<br>（檢出限 α-六六六 1.61μg/kg, β-六六六 2.96μg/kg, δ-六六六 1.43μg/kg<br>LOD α- HCH 1.61μg/kg, β- HCH 2.96μg/kg, δ- HCH 1.43μg/kg）	未檢出 Not detected<br>符合規定 Conform
- 林丹 Lindane	≤0.6 mg/kg<br>（檢出限 LOD 1.56μg/kg）	未檢出 Not detected<br>符合規定 Conform
- 五氯硝基苯 Quintozene	五氯硝基苯，五氯苯胺及甲基五氯硫基苯之和≤1.0 毫克/千克<br>Sum of quintozene, pentachloroaniline and methyl pentachlorophenyl sulphide≤1.0 mg/kg<br>（檢出限五氯硝基苯 1.92μg/kg, 五氯苯胺 1.59μg/kg, 甲基五氯硫基苯 1.33μg/kg<br>LOD quintozene 1.92μg/kg , pentachloroaniline 1.59μg/kg , methyl pentachlorophenyl sulphide 1.33μg/kg）	未檢出 Not detected<br>符合規定 Conform
8. 微生物限度#<br>Microbial Limit		
-需氧菌總數 TAMC (Total Aerobic Microbial Count)	≤500cfu/g	100cfu/g<br>符合規定 Conform
-霉菌和酵母菌總數 TYMC (Total Yeast and Mold Microbial Count)	≤100cfu/g	＜10cfu/g<br>符合規定 Conform
- 大腸埃希菌 Escherichia Coli	不得檢出/克 Not detected/g	未檢出/克 Not detected/g<br>符合規定 Conform

****

編制 Report compiled by：[signature]　　審核 Reviewed by：[signature]

培力（南寧）藥業有限公司檢測中心 PuraPharm (Nanning) Pharmaceuticals Co.,Ltd. Testing Laboratory
郵編(Postal No.)：530007　電話(Tel)：0771-3218026　傳真(Fax)：0771-3216602

註：Remarks

1、 報告無"檢驗/檢測報告專用章"和缺騎縫章無效。

Test report without the stamp of "For Test/Test Report Only" is not authorized.

2、 複製的報告未重新加蓋"檢驗/檢測報告專用章"及騎縫章無效。

Certified copy of test reportwithout the stamp of"For Test/Test Report Only" is not authorized.

3、 報告無編制、審核及本中心代表簽字無效。

Test report must be authorized by the signature of the bodies of preparation, verification and representative of the testing centre.

4、 報告塗改、缺頁無效。

Test report with modification or lacking of page(s) is not authorized.

5、 對報告若有異議，請於收到報告之日起十五日內向檢驗單位提出書面申訴，否則按認可檢驗報告處理。本中心異議受理電話 0771-3218026。

The client is aggrieved by the test result. A request for review shall state in writing the reasons relied upon and shall be made to the testing centre within 15 days after receipt of the authorized test report. No requirement will be accepted after the definite time. Inquiry hot line is 0771-3218026.

6、 送樣委託檢驗，樣品名稱為委託單位自報名稱，報告僅對來樣負責。部份複製檢驗/檢測報告無效。

Name of tested product is provided by the client. The report will refer only to the sample tested. Copy of partial of the test report is not authorized.

# - 其他微生物限度要求: Other requirements for Microbial Limit Test

1. 含動物類藥材（包括提取物）的中藥固體製劑，每 10g 不得檢出沙門菌。

TCM solid preparation containing animal raw materials (including extract): Absence of Salmonella (10g).

2. 含藥材原粉的中藥固體製劑，每 10g 不得檢出沙門菌；耐膽鹽革蘭陰性菌應小於 10^2cfu（1g）。

TCM solid preparation containing herbal raw powders: Absence of Salmonella (10g); Not more than 10^2cfu of Bile-tolerant gram-negative bacteria (1g).

*** End of Report 報告完 ***

营业执照

Business license

（副本） Copy

(1-1)

统一社会信用代码914501007114025671

Unified social credit code 914501007114025671

名称 培力（南宁）药业有限公司

Name:PuraPharm(Naning)Pharmaceuticals Co.,Ltd.

类型 有限责任公司(外国法人独资)

Type:Limited liability company (sole foreign-funded legal person)

住所 南宁高新区科园大道46号

Adress:No. 46, Keyuan Avenue, Nanning High-tech Zone

法定代表人 陈宇龄

Legal representative:Yu-ling Chen

注册资本 壹亿柒仟万人民币元整

Registered capital:17800 ten thousand RMB

成立日期 1998年08月19日

Register date:19th,August,1998

营业期限 1998年08月19日至2048年08月18日

Business term:From 19th,August,1998to 18th,August,2048

经营范围 生产销售片剂、颗粒剂、丸剂（小蜜丸、水丸）（含中药前处理和提取）、中药饮片（净制、切制）、中药配方颗粒（涉及国家专项管理的按照有关规定办理）（药品生产许可证有效期至２０20年１２月３１日）；进口分销保健食品（批发零售：保健食品，凭许可证开展经营活动）（凡涉及配额、许可证、专项管理的商品，按国家专项管理规定办理）；商务信息咨询服务。（依法须经批准的项目，经相关部门批准后方可开展经营活动。）

Business permission:Production and sales of tablets, granules, pills (Xiaomi Pills, Water Pills) (including pre-treatment and extraction of Chinese medicines), Chinese Herbal Pieces (net system, cut system), and Chinese medicine formula granules (regarding relevant state regulations for special management of the country) (The pharmaceutical production license is valid until December 31, 2020; import and distribution of health foods (wholesale and retail: health foods, with licenses to carry out business activities) (for commodities involving quotas, licenses, and special management, according to the national special management regulations For business); business information consulting services. (Projects that are subject to approval according to law may not be operated until approved by the relevant department.)

企业信用信息公示系统网址: http://www.gxqyxygs.gov.cn

中华人民共和国国家工商行政管理总局监制

中华人民共和国
药品生产许可证
(副本)

The people's Republic of China Drug production license

企业名称：培力（南宁）药业有限公司 Comparny Name :PuraPharm(Naning)Pharmaceuticals Co.,Ltd.

注册地址：南宁高新区科园大道46号 Registered Address:No. 46, Keyuan Avenue, High-tech Development Zone, Nanning

社会信用代码：71140256-7 Unified social credit code:71140256-7

法定代表人：陈宇龄 LegalRepresentative:Yu-ling Chen

企业负责人：陈宇龄 Business person in charge:Yu-ling Chen

质量负责人：覃艳霞 Quality Directer:Yan-xia Tan

有效期至：2020年12月31日 Period of validity:31th,December,2020

日常监管机构：南宁市食品药品监督管理局、南宁高新区食品药品监督管理局

Daly Regulators:Nanning Food and Drug Administration, Food and Drug Administration of High-tech Zone, Nanning City

日常监管人员：唐敏玲、彭琇彦、翁军、黄瑊、冯良、刘惠杰

Daly Supervisor:Min-ling Tang;XIU-yan Peng;Jun Weng;Xian Huang;Liang Feng;Hui-jie Liu

监督举报电话：12331

Supervinsion and reporting number:12331

编号：桂20160088 Number:Gui20160088

分类码：HbZbXY Classification code:HbZbXY

生产地址和生产范围：Production address and permission

广西南宁市高新技术开发区科园大道46号：片剂，颗粒剂(含中药配方颗粒)，丸剂(小蜜丸、水丸)，中药前处理和提取，中药饮片(净制、切制)***

No. 46, Keyuan Avenue, High-tech Development Zone, Nanning, Guangxi province,China.
Tablets, granules (including Chinese medicine formula granules), pills (Xiaomi Pills, water pills), pretreatment and extraction of Chinese medicines, Chinese Herbal Pieces (net system, cut system).

Lssuing authority:Guangxi Zhuang Autonomous Region Food and Drug Administration

发证机关：广西壮族自治区食品药品监督管理局

签发人：文东旭 The issuer:Dong-xu Wen

2016年01月01日 1st,Jan,2016

Change log

变 更 记 录

事项：

仓库地址：

1. 南宁市高新区振华路5号综合楼4-5层

2. 南宁市高新区总部路3号中国一东盟企业总部基地二期8号楼海格物流仓库一楼至四楼库房

Matters: Warehouse Address: 1. Floor 4-5, Complex Building, 5 Zhenhua Road, Gaoxin District, Nanning; 2. First Floor, Hager Logistics Warehouse, Building 8, China-ASEAN Corporate Headquarters, No. 3 Headquarters, High-Tech Zone, Nanning To the fourth floor warehouse

（盖章）

年 月 日

事项：同意该企业《药品生产许可证》社会信用代码由71140256-7变更为91450100711402567l；其他内容不变。

Matters: It is agreed that the social credit code of the “Drug Production License” of the enterprise will be changed from 71140256-7 to 914550110011402567l; other contents will remain unchanged.

（盖章）

2016 年 02 月 01 日

事项：同意该企业《药品生产许可证》生产地址和范围由广西南宁市高新技术开发区科园大道46号：颗粒剂(含中药配方颗粒)，片剂，丸剂(小蜜丸、水丸)，中药前处理和提取，中药饮片(净制、切制)***1.南宁市高新区振华路5号综合楼4-5层：仓库***南宁市高新区总部路3号中国—东盟企业总部基地二期8号楼海格物流仓库一楼至四楼库房：仓库***变更为广西南宁市高新技术开发区科园大道46号：颗粒剂(含中药配方颗粒)，片剂。

（盖章）

2016 年 08 月 15 日

Matters: It is agreed that the production address and scope of the “Drug Production Licence” of the company will be from No. 46, Keyuan Avenue, High-tech Development Zone, Nanning City, Guangxi Province: granules (including Chinese medicine formula granules), tablets, and pills (Xiaomi Pill, Shuiwan) , Pre-treatment and extraction of traditional Chinese medicine, Chinese Herbal Medicine (net system, cut system). ***1. Floor 4-5, Comprehensive Building, No. 5, Zhenhua Road, High-tech Zone, Nanning: Warehouse *** First Floor, Hager Logistics Warehouse, Building 8, China-ASEAN Corporate Headquarters, No. 3 Headquarters, High-tech Zone, Nanning City To the Treasury on the fourth floor: Warehouse *** was changed to No. 46, Keyuan Avenue, High-tech Development Zone, Nanning, Guangxi Province: granules (including Chinese medicine formula granules) and tablets.

事项：同意该企业《药品生产许可证》核增生产地址和生产范围为：南宁市高新区总部路3号中国-东盟企业总部基地二期3号厂房三、四层：仓库***；其他内容不变。

（盖章）

2016 年 08 月 15 日

Matters: Agreed to the company's "Drug Production License" nuclear production address and production scope: 3rd and 4th floors of No. 3 plant of China-ASEAN Enterprise Headquarters, No. 3 Headquarters Road, High-tech Zone, Nanning, China: warehouse ***; other content constant.

Change log

变 更 记 录

事项：同意该企业《药品生产许可证》质量负责人由覃艳霞变更为任翔；其他内容不变。

Matters: The person in charge of the quality of the “Drug Production License” of the company was changed from Yan-xia Tan to Xiang Ran; the other contents remain unchanged.

（盖章）

2017 年 06 月 12 日

事项：同意该企业质量负责人由任翔变更为吴振相；其他内容不变。

Matters: It is agreed that the person in charge of the quality of the company will be changed from Xiang Ran Zhen-xiang Wu; the other contents will remain unchanged.

（盖章）

2018 年 03 月 22 日

事项：

（盖章）

年 月 日
